# In Memoriam of Edward H. Koo, MD 1954–2025

**DOI:** 10.1186/s13024-025-00862-9

**Published:** 2025-06-16

**Authors:** Hui Zheng, Douglas Galasko, Sangram S. Sisodia

**Affiliations:** 1 Huffington Center On Aging, Baylor College of Medicine, Houston, TX US; 2 Department of Neurosciences, University of California San Diego, San Diego, CA US; 3 Departments of Neurobiology and Neurology, University of Chicago, Chicago, IL US


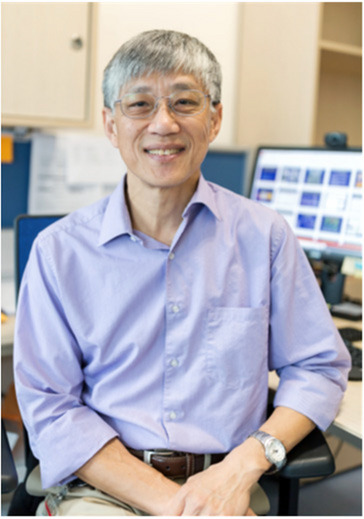
Edward H. Koo, MD, left us on April 22, 2025. At the age of 70, he was gone too soon, but he lived a full life and left a lasting legacy: He was a devoted husband to his wife Nancy, a loving father to his son Jeremy and daughter Allison (both followed his path to Amherst College), an accomplished scientist, and a celebrated mentor. To many of us, he was an extraordinary human being, a wonderful colleague, and a good friend who we fondly called Eddie.

Eddie had a distinguished career that spans over four decades. He received his Bachelor’s degree from Amherst College in 1976, a MD from Duke University School of Medicine in 1980, where he also completed residency training in pathology. In 1982, he went on to pursue a neurology residency at the University of California, San Francisco, and served as Chief Resident from 1984 to 1985. He further specialized in neuropathology through a residency at the Johns Hopkins University School of Medicine, where he also held a research and clinical fellowship and later promoted to a faculty in the Departments of Pathology and Neurology. In 1991, Eddie joined Harvard Medical School as an Assistant Professor of Pathology and Associate Neurologist and Neuropathologist at Brigham and Women’s Hospital. In 1996, he transitioned to the University of California, San Diego (UCSD), where he held faculty appointments in the Department of Neurosciences, achieving full professorship in 2000. From 2013 to 2019, he was a Professor in the Departments of Medicine and Physiology at National University of Singapore’s Yong Loo Lin School of Medicine.

During his extensive clinical training, Eddie found his passion in research and made himself a life-long cell and molecular biologist devoted to understanding the fundamental processes leading to Alzheimer’s disease. Among his many accomplishments, his pioneering work on the trafficking and processing of the amyloid precursor protein (APP) was instrumental in our understanding of how β-amyloid peptides are generated and deposited in Alzheimer’s disease. He made seminal discoveries on the function and regulation of the presenilins, mutations of which are causal for familial early onset Alzheimer’s disease. And he identified a class of molecules with the unique property to modulate gamma-secretase activity such that they curb β-amyloid peptide production without affecting other presenilin substrates. These gamma-secretase modulators are being evaluated as potential therapy for Alzheimer’s disease.

Eddie’s stellar academic record and groundbreaking research earned him numerous awards. Among others, he was elected to Phi Beta Kappa in 1976, recognizing early academic excellence. In 1981, he received the Irwin A. Brody Neuroscience Scholar Award at Duke University, followed by the Sandoz Award for Outstanding Neurology Resident at UCSF in 1985, and the William F. Milton Fund Award from Harvard University in 1992. As a junior investigator, Eddie was honored with the NIH Leadership and Excellence in Alzheimer’s Disease (LEAD) Award, and was named a Paul Beeson Physician Faculty Scholar in Aging Research by the American Federation for Aging Research. He was a recipient of the Faculty Scholar Award (1991–1994), the Zenith Award (1994–1996), and the Khalid Iqbal Lifetime Achievement Award (2017) from the Alzheimer’s Association. In 1998, he was honored with the AlliedSignal Award for Research in Aging. The following year, he was elected as a Fellow of the American Neurological Association. In 2009, Eddie received the MetLife Foundation Award for Medical Research, and in 2015, he was elected as a Fellow of the American Association for the Advancement of Science.

We were fortunate to have known Eddie for many years. Here we offer personal accounts of what Eddie meant to us.

## Sam Sisodia

I met Eddie in the late 80’s when I was a postdoc with Don Cleveland and he was a resident in Neuropath. Eddie would come over to our lab to run Northern blots and we soon became good friends. Eddie urged me to consider taking a position as a Research associate with Don Price to guide studies focused on the molecular and cellular basis of Alzheimer’s disease. Not long thereafter, I joined the Price group with Eddie serving as my mentor. With his prodigious training in neurology and neuropath, Eddie guided me through the mysteries of the brain and the challenges of understanding an enormously complex disease. Eddie and I immediately began collaborating on various aspects of APP metabolism, RNA expression and axonal trafficking, work that culminated in several papers on which we shared coauthorship. During this time, Eddie introduced me to some of the finer things in life, including red wine, sushi and grilled leg of lamb. My wife and I soon became friends with Eddie and Nancy and visited them on occasion in Columbia. My moments spent with Eddie in the lab or at our homes were some of my most revered experiences. Sadly, only two years later, Eddie chose to join the laboratory of Dennis Selkoe at Harvard. We remained good friends, but did not collaborate further. Eddie played an absolutely indispensable role in my career development and I am forever reminded about his kindness, generosity and patience throughout that journey.

## Hui Zheng

I got to know Eddie about 30 years ago. I was working at Merck Research Laboratories at the time and my first project related to AD was to create a mouse line deficient in *App*. Eddie just started his research program on APP cell biology at Brigham and Women’s Hospital and Harvard Medical School, and he was interested in learning how neurons behaved without APP. Thus, I provided the *App* knockout mice to him, and this marked the beginning of our long-standing collaboration and friendship. It was immediately clear that Eddie was exceptionally smart and knowledgeable. As someone new to AD, I learned so much from Eddie through our interactions. As a result, we co-wrote a review article titled “The amyloid precursor protein: Beyond amyloid” in Molecular Neurodegeneration in 2006. The main message is to inform readers that Aβ is one of the several APP cleavage products that is generated in a highly regulated manner, calling for the importance to understand APP trafficking, processing, and function. This review was highly cited, and we followed up with an updated review in 2011.

Building on our complementary expertise in cell biology and mouse models, our collaboration further extended to presenilins. Using a genetic rescue system, we found that loss of presenilins in the skin results in epithelial hyperplasia and skin tumorigenesis. Eddie’s group, already moved to USCD, identified a role of presenilins in β-catenin signaling known to be associated with skin tumor formation. Thus, we partnered up and initiated a series of experiments to establish the connections between the in vitro and in vivo findings. This work was significant as presenilin gamma-secretase inhibitors were being evaluated in clinical trials for blocking Aβ production. The trial was stopped, partially due to skin complications.

Our collaboration was both productive and enjoyable. Eddie became a trusted advisor and mentor, helping me build my own research program and career. Indeed, I found myself to follow his path: I joined the NIA-N review committee when he was Chair and succeeded him after he rotated off. We both served on the BrightFocus Scientific Review Committee—he as Chair, I as a member—and when he retired in 2017, I became Co-Chair. Eddie, I hope I lived up to your expectation.

Eddie endured health challenges in the last two decades of his life, yet he faced them with remarkable optimism, courage, and with a sense of humor. He became an expert on his cancer type and continued following Alzheimer’s research to the very end. Just this February, when I spoke at the Alder Symposium in San Diego, he wrote to say he wished he could attend, but his legs were too swollen to sit still.

But above all, Eddie was a kind soul and a true friend. We had many life-enriching conversations over the years—about science, careers, family, and life, and I only wish they could have continued.

## Doug Galasko

When I was a Neurology Resident, I first met Eddie during his time with Don Price’s lab at Hopkins. I followed his scientific trajectory with interest, as I became an Alzheimer’s researcher, and was highly enthusiastic when Leon Thal recruited him to UCSD in 1996. Eddie contributed to and enhanced our AD research ecosystem at UCSD in many different ways, including his stellar research, vision, collaborative nature and warm and engaging character. In addition to his laboratory’s productivity in furthering our knowledge of the intricacies of APP processing and its roles in AD, Eddie was instrumental in obtaining funding for a T32 training grant on Neuroplasticity in Aging, which included researcher/mentors from UCSD, the Salk Institute and Scripps Research Institute. Shortly after he had signed on to move to UCSD, Eddie helped us to weather the untimely passing of Tsunao Saitoh by taking over the management of laboratory funding and projects and organizing and ensuring a happy landing for staff and trainees. Similarly, after the sudden death of Leon Thal in 2007, Eddie provided remarkable leadership and support in stabilizing and reorganizing research programs and comforting people. Among his many scientific contributions while at UCSD was the finding, together with Todd Golde, that it was possible to modulate the activity of Gamma secretase. This led to P01 funding for a multi-institutional group of us to study repurposing and try to identify novel compounds as modulators and efforts by other groups. Among our own efforts, this led to a phase 1 clinical trial of R-flurbiprofen, which was taken into larger human studies. More recently, potent gamma-secretase modulators have gone through successful human clinical trials in this promising area. I benefited greatly from his warmth, support, knowledge and wisdom during this research and in our roles as co-leaders of the UCSD Alzheimer’s Disease Research Center.

I remember hearing from Eddie one fateful weekend in 2004 that a mass was found in his pancreas on a CT scan. He then went through one of the most arduous surgical procedures imaginable, and the tumor turned out to be a slower progressing neuroendocrine type, not the rapidly lethal pancreatic carcinoma. The cancer continued to grow during the 18 years but Eddie pursued science, interpersonal relationships and life with the same vitality and vigor as before. Arriving at an ADRC External Advisory Board meeting with a Pick line in place, and more recently injecting himself with insulin and carefully calibrating each meal he ate were small adaptations to continuing to travel widely and participating in advisory committees and leadership roles such as the Beeson Scholars program and BrightFocus, maintaining robust social and professional relationships, and sharing his expertise about wine, food, golf, music and life with those of us fortunate to be his colleagues, friends and family. Even in the past two years when the cancer became much more aggressive, spreading to many other areas of his body, leading to complications, his personality, warmth and expert knowledge shone through. His matter-of-fact attitude about many of these discomforts underlies his heroism.

We have cherished Eddie’s remarkable friendship, generous support, and sincere guidance—both in our careers and in life—throughout the years. It is deeply saddening, and still difficult to accept, that he is no longer with us. Yet, his kindness, wisdom, integrity, courage, and humanity continue to resonate and inspire us.

## Data Availability

No datasets were generated or analysed during the current study.

